# Tooth segmentation by low-dose CBCT for orthodontic treatment planning: Explorative ex vivo validation

**DOI:** 10.1007/s00056-024-00558-7

**Published:** 2024-10-24

**Authors:** Maurice Ruetters, Holger Gehrig, Sinclair Awounvo, Ti-Sun Kim, Sara Doll, Korallia Alexandrou, Anna Felten, Christopher Lux, Sinan Sen

**Affiliations:** 1 Heidelberg University, Department of Operative Dentistry, University Hospital Heidelberg, Im Neuenheimer Feld 400, 69120 Heidelberg, Germany; 2 University Hospital Heidelberg, Institute of Medical Biometry, Im Neuenheimer Feld 130.3, 69120 Heidelberg, Germany; 3 Department of Anatomy and Cell Biology, Heidelberg University, Im Neuenheimer Feld 307, 69120 Heidelberg, Germany; 4 Department of Orthodontics, University Hospital Heidelberg, Im Neuenheimer Feld 400, 69120 Heidelberg, Germany; 5 Department of Orthodontics, University Hospital Schleswig Holstein, Arnold-Heller-Straße 3, 24105 Kiel, Germany

**Keywords:** Tooth volume, Tooth length, Low-dose cone-beam computed tomography, Radiation exposure, Three-dimensional imaging, Zahnvolumen, Zahnlänge, Dosisreduzierte digitale Volumentomographie, Strahlenbelastung, Dreidimensionale Bildgebung

## Abstract

**Purpose:**

Three-dimensional imaging has become an increasingly important component of orthodontics. Associated with this, however, is a higher radiation exposure for patients. New cone-beam computed tomography (CBCT) devices have been developed that can provide low-dose CBCT (LD-CBCT). We hypothesized that LD-CBCT is as precise and reproducible as standard high-dose CBCT (HD-CBCT) in segmenting roots and crowns as well as measuring tooth length.

**Methods:**

HD-CBCT and LD-CBCT scans were taken of four human cadaveric heads. Thirty single-rooted teeth were segmented twice by one investigator. The length of each tooth was also measured. Lin’s concordance correlation coefficient (CCC) was calculated to assess the agreement of HD-CBCT and LD-CBCT measurements and the intraclass correlation coefficient (ICC) was calculated to assess intrarater reliability. Analyses were supported by Bland–Altman plots.

**Results:**

Volume measurements obtained using HD-CBCT were significantly higher than those obtained using LD-CBCT (*p* < 0.001). CCC was 0.975 (95% confidence interval [CI] = 0.956–0.986) indicating excellent agreement between the two modalities. Intrarater reliability between the two sets of LD-CBCT and HD-CBCT volume measurements was excellent (ICC = 0.998, 95%CI = 0.995–0.999 [HD-CBCT], ICC = 0.997, 95%CI = 0.992–0.998 [LD-CBCT]). CCC for tooth length measurements was 0.991 (95% CI = 0.983–0.995), indicating excellent agreement between HD-CBCT and LD-CBCT. Intrarater reliabilities between the two sets of tooth length measurements were also excellent for both methods (ICC = 0.998, 95%CI = 0.995–0.999 [HD-CBCT], ICC = 0.997, 95%CI = 0.992–0.998 [LD-CBCT]).

**Conclusions:**

Within the limitations of this experimental setting, LD-CBCT is as valid as HD-CBCT for measuring tooth length. Regarding the volume differences, in vivo studies are required to determine their clinical relevance.

## Abstract (de)

**Zielsetzung:**

Dreidimensionale Bildgebung ist zu einem immer wichtigeren Bestandteil der Kieferorthopädie geworden. Damit verbunden ist jedoch eine höhere Strahlenbelastung für Patienten. Neu entwickelte Geräte für die digitale Volumentomographie („cone-beam computed tomography“, CBCT) ermöglichen Tomographien mit reduzierter Strahlenbelastung („low dose cone-beam computed tomography“, LD-CBCT). Wir stellten die Hypothese auf, dass die LD-CBCT bei der Segmentierung von Zahnwurzeln und -kronen sowie bei der Messung von Zahnlängen genauso präzise und reproduzierbar ist wie die konventionelle HD-CBCT mit hoher Strahlendosis.

**Methode:**

Von 4 Köpfen menschlicher Leichen wurden HD-CBCT- und LD-CBCT-Scans angefertigt. 30 Zähne mit einer Wurzel wurden von einem Untersucher zweimal segmentiert. Die Länge jedes Zahns wurde ebenfalls gemessen. Der Konkordanz-Korrelationskoeffizient (CCC) nach Lin wurde berechnet, um die Übereinstimmung von HD-CBCT- und LD-CBCT-Messungen zu ermitteln. Zur Bewertung der Intrarater-Reliabilität wurde der Intraklassen-Korrelationskoeffizient (ICC) berechnet. Die Analysen wurden durch Bland-Altman-Diagramme bestätigt.

**Ergebnisse:**

Die mit HD-CBCT ermittelten Volumenmessungen waren signifikant höher als die mit LD-CBCT ermittelten (*p* < 0,001). Der CCC betrug 0,975 (95 %-Konfidenzintervall [KI] = 0,956–0,986), was auf eine hervorragende Übereinstimmung zwischen beiden Modalitäten hinweist. Die Intrarater-Reliabilität zwischen den beiden Sätzen von LD-CBCT- und HD-CBCT-Volumenmessungen war ausgezeichnet (ICC = 0,998, 95 %-KI = 0,995–0,999 [HD-CBCT], ICC = 0,997, 95 %-KI = 0,992–0,998 [LD-CBCT]). Die CCC für Zahnlängenmessungen betrug 0,991 (95 %-KI = 0,983–0,995), was auf eine hervorragende Übereinstimmung zwischen HD-CBCT und LD-CBCT hinweist. Auch die Intrarater-Reliabilität zwischen den beiden Messreihen der Zahnlänge war bei beiden Methoden ausgezeichnet (ICC = 0,998, 95 %-KI = 0,995–0,999 [HD-CBCT], ICC = 0,997, 95 %-KI = 0,992–0,998 [LD-CBCT]).

**Schlussfolgerungen:**

Innerhalb der Grenzen dieser experimentellen Anordnung ist LD-CBCT für die Messung der Zahnlänge genauso aussagekräftig wie HD-CBCT. Hinsichtlich der Volumenunterschiede sind In-vivo-Studien erforderlich, um ihre klinische Relevanz zu bestimmen.

## Introduction

Cone-beam computed tomography (CBCT) is a common imaging technique that enables three-dimensional visualization of structures relevant to orthodontics, such as impacted canines. CBCT can also be used to accurately describe the buccal bone adjacent to teeth [1–3]. Recent studies have used CBCT and digital scanning to predict root position after orthodontic treatment. This technique has many advantages. It may help to precisely define the marginal areas of tooth movement, including in the bucco-oral direction, preventing accidental tooth movement out of the bony envelope and unwanted gingival recessions [4–6]. This technique may also reduce the number of CBCTs required during orthodontic therapy, resulting in a reduction of radiation exposure, which is in line with the “as low as diagnostically acceptable” (ALADA) principle [7]. Reducing radiation exposure is a high priority especially in orthodontics because most patients are still very young. Younger patients have a higher lifetime radiation risk than adults because of the higher dose per mAs and the increased lifetime risk per unit dose [8]. Therefore, two-dimensional imaging modalities such as panoramic x‑rays (OPT) and cephalometrics have been routinely used to plan and monitor treatment despite their limitations because they expose patients to far less radiation than a conventional CBCT scan does [9–11]. With up to 282 mSv, conventional CBCT scans have far higher effective radiation doses for the patients than OPT does with 2.5–24.3 mSv [12–14]. However, in recent years, low-dose CBCT (LD-CBCT) protocols have been developed that allow three-dimensional imaging of dental structures with a significantly lower effective radiation dose of 12–29 mSv [13, 15]. A review of the different LD protocols found that their low radiation doses were still effective [15]. Studies have already shown that these protocols can image various structures including peri-implant defects and buccal bone lamellae in vitro [2, 16, 17].

The present explorative study investigated the ability of LD-CBCT to segment and measure teeth and dental crowns in the context of orthodontic treatment planning. We hypothesized that LD-CBCT would match the precision and reproducibility of standard high-dose CBCT (HD-CBCT) in segmenting roots and crowns as well as measuring tooth length, within this experimental design.

## Materials and methods

This ex vivo study investigated 30 single rooted teeth from four human hemisected cadaveric heads. The types of teeth included in this study are described in Table 1. The number of teeth was limited to the availability of cadaveric heads.

**Table 1:** Types of included teeth Eingeschlossene Zahnarten

Tooth type	Number
Upper front tooth	5
Upper canine	1
Upper premolar	2
Lower front tooth	11
Lower canine	5
Lower premolar	6

**Fig. 1:**
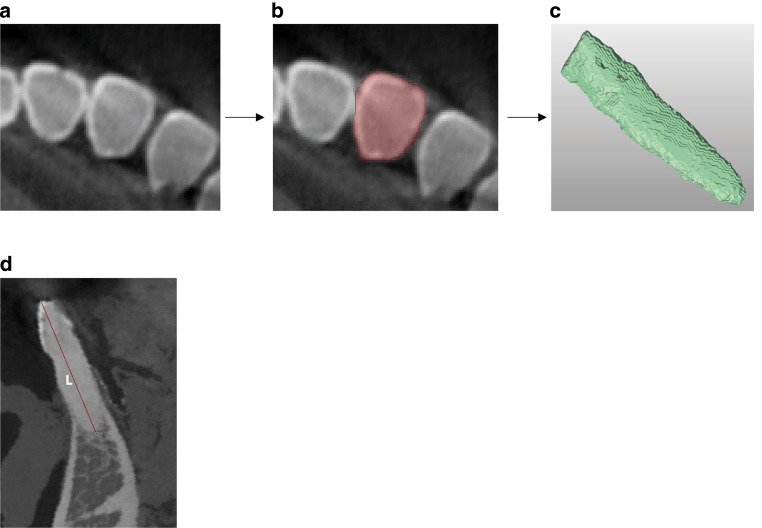
Flowchart of tooth volume (**a**–**c**) and tooth length (**d**) measurements. **a** Axial high-dose cone-beam computed tomography (HD-CBCT) slice, **b** segmentation of tooth 31 in the axial HD-CBCT slice, **c** three-dimensional model of the tooth after segmentation, **d** measurement of tooth length (L) Flowchart der Messungen von Zahnvolumen (**a–c**) und Zahnlänge (**d**). **a** Axiale Schichtaufnahme, high-dose digitale Volumentomographie (HD-CBCT), **b** Segmentierung von Zahn 31 in der axialen HD-CBCT-Schicht, **c** dreidimensionales Modell des Zahns nach der Segmentierung, **d** Messung der Zahnlänge (L)

In accordance with the ethical approval, cadaveric heads were from bodies donated to the Institute of Anatomy and Cell Biology of the University of Heidelberg and were preserved with 99% ethanol and glycerin and 37% formalin. Teeth had to have an existing crown and had to be free of extensive restorations. At the time of the radiographic investigations, the hemisected cadaveric heads, including the mandibles, were fully covered by soft tissue and adjacent cheek muscles. The tongue, neck muscles, skull base, and cervical vertebrae were also present. Each tooth was radiographically imaged in the same position using LD-CBCT and HD-CBCT (Fig. 1) on one device (Orthophos 3D SL, Dentsply Sirona, Bensheim, Hessen, Germany).

The volumetric acquisition protocols were as follows:

- LD-CBCT protocol: radiation time 2.1 s, 10 mA, 85 kV, FOV 8 × 8 cm^2^, isotropic voxel size 0.16 mm, DAP 69 mGy cm^2^, CDTI16 0.27 mGy.
- HD-CBCT protocol: radiation time 14.2 s, 6 mA, 85 kV, FOV 8 × 8 cm^2^, isotropic voxel size 0.08 mm, DAP 943 mGy cm^2^, CDTI16 4.0 mGy.

## Image review

CBCT data were exported in DICOM format to a medical image processing software (The Medical Imaging Interaction Toolkit [MITK] workbench v2021.02 [ITK 4.13.3/VTK 9.0.1/Qt 5.12.10], German Cancer Research Center [DKFZ], Heidelberg, Baden–Württemberg, Germany) for analysis. Windowing and leveling were allowed. Evaluations were all performed on a certified monitor (RadiForce RS 210, EIZO Europe GmbH, Mönchengladbach, North Rhine–Westphalia, Germany) in the same dark room.

Teeth were fully segmented semi-automatically with the work bench autosegmentation tool in LD-CBCT and HD-CBCT scans by one dentist with more than 9 years of experience (MR) in a standardized procedure: First, an automatic tool of the work bench was applied in the initial step. In the second step, this was checked in each axial layer and manually adjusted if necessary. The review was carried out from coronal to apical. Afterwards, data were stored as standard tessellation language (STL) files and exported to a 3D-Reverse-Engineering-Software (Geomagic Design X software, 3D Systems, Rock Hill, SC, USA) to calculate the volume of each tooth (Fig. 1a–c).

In respect of root resorptions, morphology, and root topography for treatment planning, tooth lengths from the most apical point to the most coronal point in one sagittal slice were also measured by the same investigator (MR; Fig. 1d) to validate the linear measurements in both protocols. Measurements were repeated after one month by the same rater (MR) to determine the intrarater reliability. The rater was blinded to the first measurements and there was a 2-week interval between LD-CBCT and HD-CBCT measurements to avoid a memory effect.

To calibrate the method, measurements and segmentations were taken of 20 teeth from different CBCT datasets and discussed with a second rater (HG) with more than 16 years of experience in CBCT diagnostics until consensus was reached.

## Statistical analysis

In this study, the measures of interest and, thus, the primary outcomes were the tooth volume and tooth length.

All metric variables in the dataset were described numerically using minimum, maximum, mean ± standard deviation (SD), first (Q1) and third quartiles (Q3). Dependently on whether the intermethod or the method reliability were investigated, variables were described separately by trial or depending on the dosis type, respectively.

To investigate whether the measurements obtained using LD-CBCT as opposed to using HD-CBCT significantly differed on average, a Wilcoxon two-sample signed-rank test was performed for each trial and outcome. In addition, Lin’s concordance correlation coefficient (CCC) alongside with 95% confidence intervals (CI) were calculated for each outcome to assess the extent of agreement of the measurements obtained using HD- and LD-CBCT. Bland–Altman plots were also drawn to support the analysis of the measurements’ agreement.

Analogously, for a given dosis type (HD- or LD-CBCT), a Wilcoxon two-sample signed-rank test was used to assess, separately for each outcome, whether measurements obtained at the two trials significantly differed on average. Further, depending on the dosis type, the intraclass correlation coefficient alongside 95% CI was calculated and Bland–Altman plots were drawn to investigate the strength of the agreement between the measurements at the two trials.

All statistical analyses were performed using two software environments for statistical computing and graphics (R version 4.2.1., The R Foundation, Vienna, Austria and SPSS IBM Statistics version 27.0.0.0, IBM, Armonk, NY, USA).

## Results

### Tooth volume

As described, the segmentation of the teeth in both protocols was carried out twice by the same examiner (MR). The descriptive results are shown in Table 2.

**Table 2:** Low-dose cone-beam computed tomography (LD-CBCT) and high-dose CBCT (HD-CBCT) tooth volume measurements Zahnvolumenmessungen mit dosisreduzierter digitaler Volumentomographie (LD-CBCT) und high-dose digitaler Volumentomographie (HD-CBCT)

Variable	HD-CBCT (*N* = 30)	LD-CBCT (*N* = 30)	*p*
*Volume measurement 1 (mm*^*3*^*)*			
*N*	30	30	< 0.001
Mean ± SD	352.51 ± 126.01	326.49 ± 115.17	
Median	344.9	309.1	
Q1–Q3	264.3–425	242.9–398.7	
Min–max	173–634	163–584	
*Volume measurement 2 (mm*^*3*^*)*			
*N*	30	30	< 0.001
Mean ± SD	352.97 ± 126.7	331.23 ± 119.27	
Median	340.2	333.25	
Q1–Q3	263–420.7	239.5–391.1	
Min–max	168–639	160–610	

*SD* standard deviation, *min* minimum, *max* maximum, *Q1, Q3* first, third quarter, *measurement 1 *first round of measurements, *measurement 2* second round of measurements

**Fig. 2:**
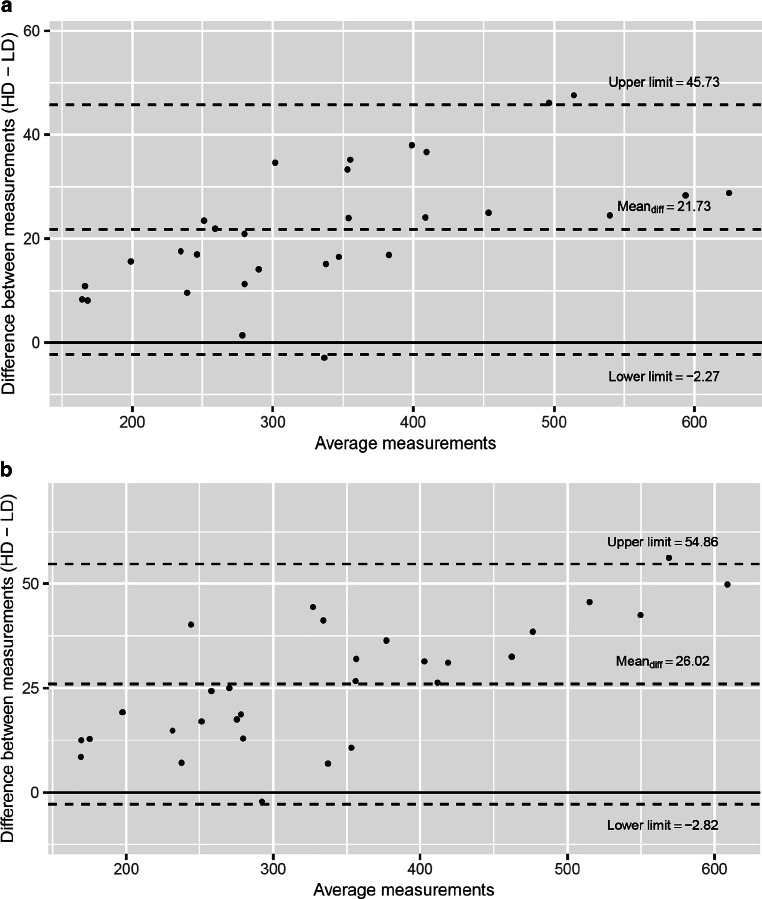
Bland–Altman plots of low-dose (LD-CBCT) and high-dose cone-beam computed tomography (HD-CBCT) volume measurements (mm^3^) from **a** the first round of measurements and **b** the second round of measurements Bland-Altman-Plots der Zahnvolumenmessungen (mm^3^) mit der dosisreduzierten (LD-CBCT) und der high-dose digitalen Volumentomographie (HD-CBCT) **a** aus der ersten und **b** aus der zweiten Messrunde

Means were 352.51 mm^3^ (HD-CBCT 1)/352.97 mm^3^ (HD-CBCT 2) versus 326.49 mm^3^ (LD-CBCT 1)/331.23 mm^3^ (LD-CBCT 2). The differences between HD-CBCT and LD-CBCT were significant (*p* < 0.001) for both segmentation rounds (1 and 2). In both segmentation rounds, the average volume in HD-CBCT was larger than in LD-CBCT (Fig. 2).

The means of the differences, illustrated in the Bland–Altman plots were 21.7 mm^3^ (segmentation round 1) and 26.02 mm^3^ (segmentation round 2). The Lin’s CCC was 0.975 (95% CI = 0.956–0.986), indicating excellent agreement between LD-CBCT and HD-CBCT tooth volume measurements [18]. The intrarater reliabilities were also excellent between the two rounds of segmentation of tooth volumes in HD-CBCT as well as LD-CBCT (ICC = 0.998, 95% CI = 0.995–0.999 [HD-CBCT]; ICC = 0.997, 95% CI = 0.992–0.998 [LD-CBCT]) indicating high reliabilities of both protocols [18].

### Tooth length

Analogous to the segmentations, tooth length measurements were also performed twice by the same examiner (MR). The results are presented in Table 3.

**Table 3:** High-dose (HD-CBCT) and low-dose cone-beam computed tomography (LD-CBCT) tooth length measurements Zahnvolumenmessungen mit high-dose digitaler Volumentomographie (HD-CBCT) und mit dosisreduzierter digitaler Volumentomographie (LD-CBCT)

Variable	HD-CBCT (*N* = 30)	LD-CBCT (*N* = 30)	*p*
*Length measurement 1 (mm)*			
*N*	30	30	< 0.586
Mean ± SD	20.93 ± 2.912	20.95 ± 2.803	
Median	21.24	21.30	
Q1–Q3	18.73–22.87	18.95–22.76	
Min–max	14–27	15–27	
*Length measurement 2 (mm)*			
*N*	30	30	< 0.059
Mean ± SD	20.89 ± 2.948	21.04 ± 2.837	
Median	21.28	21.40	
Q1–Q3	18.56–22.90	18.76–22.89	
Min–max	14–27	14–28	

*SD* standard deviation, *min* minimum, *max* maximum, *Q1, Q3* first, third quarter, *measurement 1 *first round of measurements, *measurement 2* second round of measurements

**Fig. 3:**
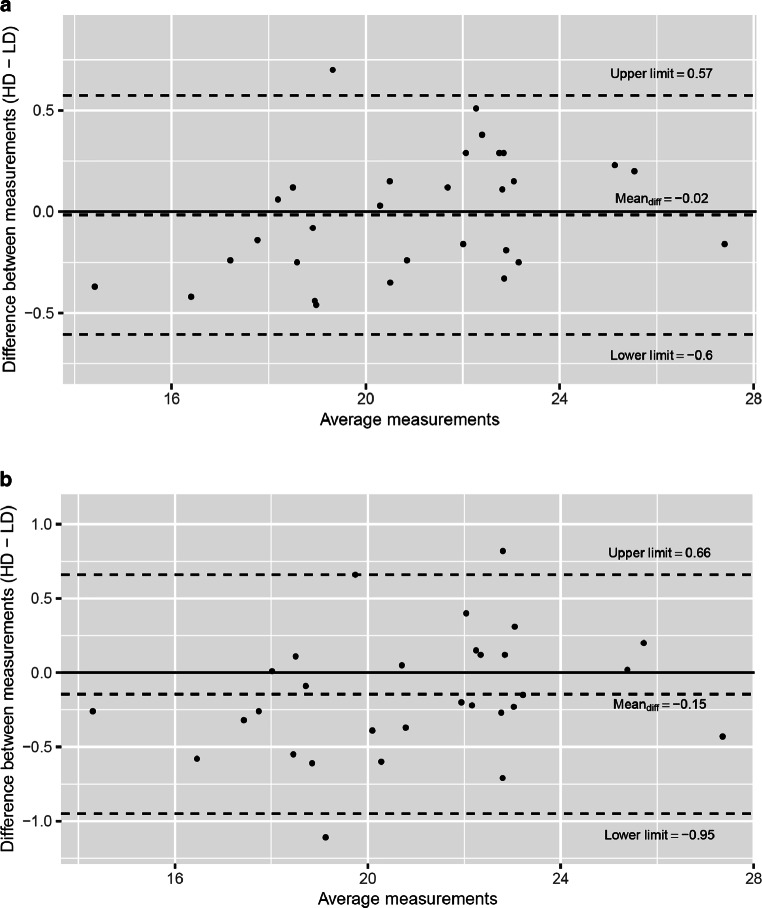
Bland–Altman plots of low-dose (LD-CBDT) and high-dose cone-beam computed tomography (HD-CBCT) tooth length measurements (mm) from **a** the first round of measurements and **b** the second round of measurements Bland-Altman-Plots der Zahnlängenmessungen (mm) mit der dosisreduzierten (LD-) und der high-dose digitalen Volumentomographie (HD-CBCT) **a** aus der ersten und **b** aus der zweiten Messrunde

Means were 20.93 mm (HD-CBCT 1)/21.28 mm (HD-CBCT 2) versus 20.95 mm (LD-CBCT 1)/21.04 mm (LD-CBCT 2). The differences between HD-CBCT and LD-CBCT were not significant (*p* = 0.586 [round 1], *p* = 0.049 [round 2]). The means of the differences, illustrated in the Bland–Altman plots were −0.02 mm (round 1) and −0.15 mm (round 2; Fig. 3).

The Lin’s CCC was 0.991 (95% CI = 0.983–0.995), indicating excellent agreement between LD-CBCT and HD-CBCT tooth length measurements [19]. The intrarater reliabilities between the two sets of LD-CBCT and HD-CBCT tooth length measurements were also excellent (ICC = 0.998, 95% CI = 0.995–0.999 [HD-CBCT], ICC = 0.997, 95% CI = 0.992–0.998 [LD-CBCT]) [18].

## Discussion

The results of our study partly confirmed the hypothesis that LD-CBCT would match the precision and reproducibility of standard HD-CBCT in segmenting roots and crowns and measuring tooth lengths, within the chosen experimental design.

Our results confirm that LD-CBCT is as suitable as HD-CBCT for measuring tooth length. High Lin’s CCC values (> 0.9) showed that LD-CBCT has the same potential as HD-CBCT to measure apical–occlusal tooth length. Furthermore, high ICC values (> 0.9) indicated that these measurements were highly reproducible in both LD-CBCT and HD-CBCT and that the anatomic tooth landmarks (apex of the root and occlusal part of the crown) were clearly identifiable on LD-CBCT and HD-CBCT. *P*-values showed no statistically significant difference between the measurements in both protocols. These results are in line with those of other studies addressing the suitability of LD-CBCT for imaging dental structures and showing that LD-CBCT has great potential to visualize periodontal and peri-implant morphology [2, 17, 20].

Concerning segmentation of the crowns and roots, the hypothesis could not fully be confirmed. There were also high values for Lin’s CCC and ICC (> 0.9) indicating valid and reliable results. But the volumes showed significant differences (*p* < 0.001). In line with these results, Bland–Altman plots revealed that HD-CBCT resulted in higher volume values. However, one must consider that the *p*-values can only be interpreted with their descriptive character due to the explorative character of this study. Thus, it is unclear whether these differences are of clinical relevance.

The results of the present study are relevant to orthodontic treatment. Three-dimensional imaging allows more predictable therapy with fewer side effects and lower radiation exposure [4–6]. CBCT combined with oral scans can predict exact tooth positions, thereby avoiding undesirable side effects such as gingival recessions following inadequate tooth movements out of the bony envelope. Moreover, promising results for automated segmentation by HD-CBCT have already been shown by a study, which demonstrated that automated segmentation using artificial intelligence was as good as semi-automated segmentation [21]. Evidence concerning these use cases are still restricted to conventional HD-CBCT protocols. The utilization of LD-CBCT protocols could, therefore, contribute to achieving a significant radiation dose reduction for the often very young patients in orthodontics.

### Limitations

Limitations of this study must be mentioned. The ex vivo nature of the experiments eliminated the risk of natural motion such as tremors, which can lead to motion artifacts that significantly reduce the quality and information content of the image [22, 23]. Furthermore, none of the included teeth had metallic restorations, which can also cause imaging artifacts that interfere with proper visualization of tooth edges [24]. However, this is representative of orthodontic patients, who are often young children without restorations [25].

Another limitation of the study is that only half heads were used. Gel pads were used to mimic the missing half, but these cannot fully imitate natural bony structures, teeth, restorative materials, or the artifacts caused by these structures. This means that the image quality may have been slightly better than it would have been for complete heads [26].

## Conclusion

Within the limitations of this experimental setting, low-dose cone-beam computed tomography (LD-CBCT) is as valid as high-dose CBCT for measuring tooth length. Regarding the observed volume differences, in vivo studies are required to determine their clinical relevance.

## Data Availability

The data supporting the conclusions of this article will be made available by the authors on request.
